# Cardiac safety analysis of anti-HER2-targeted therapy in early breast cancer

**DOI:** 10.1038/s41598-022-18342-1

**Published:** 2022-08-22

**Authors:** Li Zhang, Yan Wang, Wenjing Meng, Weipeng Zhao, Zhongsheng Tong

**Affiliations:** 1 Department of Breast Oncology, Tianjin Medical University Cancer Institute and Hospital, National Clinical Research Center for Cancer, West Huanhu Road, Tianjin, 300060 People’s Republic of China; 2 Key Laboratory of Breast Cancer Prevention and Treatment, Tianjin Medical University, Ministry of Education, Tianjin’ Clinical Research Center for Cancer, Tianjin, 300060 People’s Republic of China

**Keywords:** Breast cancer, Heart failure

## Abstract

To evaluate the cardiac safety of anti-HER2-targeted therapy for early breast cancer; to investigate whether trastuzumab combined with pertuzumab increases cardiac toxicity compared with trastuzumab; to evaluate the predictive value of high-sensitivity Troponin (hs-TnI) and QTc for the cardiotoxicity associated with anti-HER2 targeted therapy in early breast cancer. A total of 420 patients with early-stage HER2-positive breast cancer who received trastuzumab or trastuzumab combined with pertuzumab for more than half a year in Tianjin Medical University Cancer Hospital from January 2018 to February 2021 were included. Left ventricle ejection fraction (LVEF), hs-TnI values, and QTc were measured at baseline and 3, 6, 9, 12 months. Cardiotoxicity was defined as a decrease in LVEF of at least 10 percentage points from baseline on follow-up echocardiography. Cardiotoxicity developed in 67 of the 420 patients (15.9%) and all patients had LVEF above 50% before and after treatment. The incidence of cardiotoxicity in trastuzumab and trastuzumab combined with pertuzumab was 14.3% and 17.9%, respectively (*P* > 0.05). Logistic regression analysis showed that age, coronary heart disease, left chest wall radiotherapy, and anthracyclines sequential therapy were independent risk factors for cardiotoxicity (*P* < 0.05). The value of hs-TnI and QTc at the end of treatment (12th month) were selected for ROC curve prediction analysis and the area under the ROC curve was 0.724 and 0.713, respectively, which was significantly different from the area of 0.5 (*P* < 0.05). The decrease of LVEF in the study was mostly asymptomatic, from the heart safety point of view, the anti-HER2 targeted therapy for early breast cancer was well tolerated. Trastuzumab combined with pertuzumab did not significantly increase cardiotoxicity. However, subgroup analysis suggests that in the presence of coronary artery disease (CAD) and sequential treatment with anthracene, trastuzumab and pertuzumab may increase the cardiac burden compared with trastuzumab. Hs-TnI and QTc may be useful in monitoring and predicting cardiotoxicity associated with anti-HER2 targeted therapy for early breast cancer.

## Introduction

Breast cancer is one of the most common malignancies in women^1^. Studies have shown that about 20–25% of breast cancer patients have overexpression of human epidermal growth factor receptor 2 (HER2), which is closely related to poor prognosis of patients^2^. Trastuzumab, a humanized mouse monoclonal antibody against the HER2 extracellular domain IV, has been shown to inhibit HER2 overexpression through ligand-independent isomerization. It can also bind to Fc receptors on immune effector cells to induce antibody dependent cellular cytotoxicity (ADCC) of HER2-positive tumor cells to kill tumor cells^3^. Pertuzumab is another recombinant humanized monoclonal antibody that binds to the HER2 extracellular domain II, which is located opposite to domain IV, where trastuzumab binds, the mechanism of action is complementary to trastuzumab^4^. Based on the above mechanism, the combination of the two can enhance the blocking effect on the downstream signaling pathway; At the same time, it can also play the role of ADCC together to enhance the synergistic effect of immunity^5^. In the NeoSphere and TRYPHAENA study, trastuzumab combined with pertuzumab dual-targeted therapy further improved the pathological complete response rate (PCR) in neoadjuvant treatment of early breast cancer^6,7^. APHINITY study showed that trastuzumab and pertuzumab dual targeted therapy significantly improved invasive disease free survival (iDFS) when compared with trastuzumab single target treatment^8^. This establishes the role of pertuzumab-based dual anti-HER2 therapies in neoadjuvant and adjuvant therapy for early HER2-positive breast cancer.

Some studies have shown that anti-HER2-targeted therapy may increase the risk of cardiovascular toxicity. The death of cardiomyocytes occurs through multiple pathways including HER2 blocking and the increase of reactive oxygen products^9–11^. Because trastuzumab and pertuzumab complement each other in the mechanism of action, dual-target combination therapy can more completely block the HER2 signaling pathway that maintains normal function on cardiomyocytes, leading to cardiomyocyte apoptosis^12^. The effect of HER2-targeted therapy-related cardiotoxicity is dose-independent and mostly reversible, which belongs to type II cardiotoxicity; it is usually manifested as an asymptomatic decrease in LVEF, with an incidence of about 5–19%, heart failure is rare, occurring in about 1–4%^13–16^, and usually occurs in combination with anthracyclines^17–21^. Therefore, given the importance of the HER2 signaling pathway in cardiomyocytes, whether the use of trastuzumab combined with pertuzumab double target therapy at the cost of increase cardiotoxicity has been of great concern.

In August 2016, the European Society of Cardiology (ESC) published the European Society of Cardiology Position Statement on Cancer Treatment and Cardiovascular Toxicity 2016, which is the first guideline document in the field of oncology Cardiology to guide clinical practice. This paper highly affirmed the value of traditional Echocardiography (ECHO) in the assessment of cardiac function injury, especially the left ventricular ejection fraction (LVEF) measured by ECHO^22^. However, many researchers believe that ECHO lacks the sensitivity to early detection of small changes in cardiac function^23^. It has been reported that hs-TnI and QTc (QT interval corrected according to heart rate) of electrocardiogram (ECG) may have high diagnostic and predictive value in reflecting the early myocardial injury of cancer patients after receiving high-dose radiotherapy and chemotherapy^24–26^. However, further analysis is needed to determine whether they have clinical value in assessing the cardiotoxicity associated with anti-HER2-targeted therapy in breast cancer. This study will comprehensively analyze and evaluate the effect of anti-HER2-targeted therapy on cardiac function in early HER2-positive breast cancer from three aspects of ECHO, cardiac biomarkers and ECG, to provide more guidance for clinical medication.

## Methods

### Study population

Patients diagnosed with early-stage HER2-positive breast cancer who received trastuzumab or trastuzumab combined with pertuzumab therapy for more than half a year from January 2018 to February 2021 in Tianjin Medical University Cancer Hospital were collected. The criteria for admission were as follows: Stage I–III breast cancer; HER2 positive; after breast cancer trastuzumab or trastuzumab combined with pertuzumab treatment for more than half a year; relatively complete clinicopathological data were obtained, including age of onset, menopausal status, tumor stage, histological grade, pathological type, hormone receptor status, Ki-67 expression level, surgical method, medication history, radiotherapy location, etc.; echo, cardiac biomarker and ECG were detected before targeted therapy, and the above tests were performed regularly every 3 months (± 2 weeks) on the day before treatment cycle; baseline LVEF ≥ 50%. Exclusion criteria: metastatic breast cancer; a history of congestive heart failure, uncontrolled ventricular arrhythmias or myocardial infarction; combined with other primary malignant tumors; incomplete clinical and pathological data or lost follow-up. Administration method of trastuzumab: a 3-week regimen with an initial load dose of 8 mg/kg, followed by 6 mg/kg for more than half a year; In weekly regimen, the initial loading dose was 4 mg/kg and then maintained at 2 mg/kg for more than half a year. Pertuzumab: A 3-week regimen with an initial dose of 840 mg and a maintenance dose of 420 mg for more than 6 months. Targeted therapies can be used in combination with chemotherapy.

### Definition

Estrogen receptor (ER), progesterone receptor (PR) and HER2 were evaluated according to the scoring system recommended by the guidelines of the American Society of Clinical Oncology (ASCO) and the Association of American Pathologists (CAP). Breast Cancer staging is based on the American Joint Committee on Cancer (AJCC) Eighth Edition TNM Staging Guidelines. Body Mass Index (BMI) = weight (kg) ÷ height^2^ (m). Corrected QT interval (QTc), QT interval refers to the time required for the whole process of ventricular depolarization and repolarization, from the beginning of QRS wave group to the end point of T wave. ${\text{QTc}} = {\text{QT}}\sqrt {{\text{RR}}} ,$ RR represents heart rate^27^. The cardiotoxicity diagnosis in this study was LVEF decreased from baseline > 10% and to a value of < 50%^22^.

### Ethics statement

Ethics Committee Approval: The authors declare that the study was conducted in accordance with the principles of the World Medical Association Declaration of Helsinki “Ethical Principles for Medical Research Involving Human Subjects” (revised in October 2013). The ethical approval for this research was obtained from the Medical Ethics Committee of Tianjin Medical University Cancer Institute and Hospital. And all patients participating in the study received written informed consent.

### Data analysis

IBM SPSS Statistics 26.0 software was used for statistical data analysis, and GraphPad Prism 8.0 was used for plotting. Enumeration data were described by frequency and percentage, while measurement data were described by mean ± standard deviation. LVEF and QTc included in the analysis were normally distributed by K–S test. Paired T test was used for comparison before and after treatment, and independent sample T test was used for comparison between groups. The K–S test did not conform to the normal distribution of hs-TnI, Wilcoxon signed rank sum test was used for comparison before and after treatment, and Mann–Whitney U test was used for comparison between groups. Logistic regression model was used to analyze univariate and multivariate cardiotoxicity associated with anti-HER2 targeted therapy in breast cancer. Receiver operating curve (ROC) was used to predict the diagnostic value of hs-TNI and QTc for the cardiotoxicity associated with anti-HER2 targeted therapy in breast cancer. All statistical results were based on *P* < 0.05 indicates that the difference is statistically significant.

## Results

**Figure 1:**
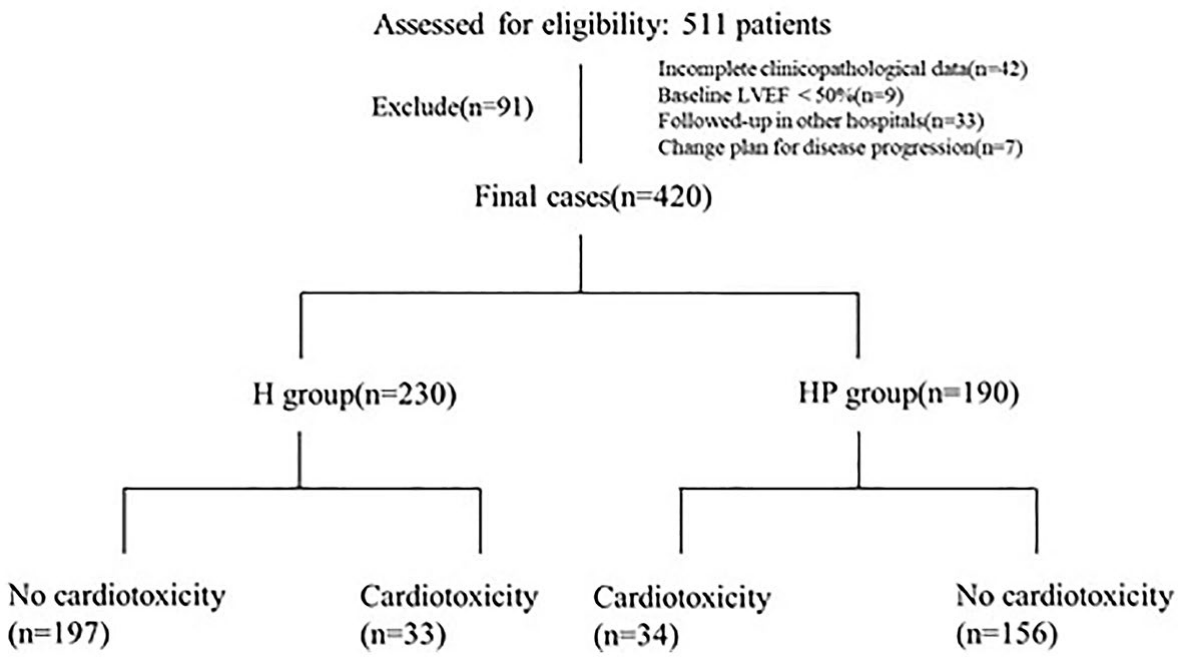
The flowchart of the study. (*H* trastuzumab, *HP* trastuzumab and pertuzumab).

From January 2018 to February 2021, a total of 511 patients receiving anti-HER2-targeted therapy for breast cancer in Tianjin Medical University Cancer Hospital were collected and screened according to inclusion and exclusion criteria. Finally, 420 patients were included in the analysis (Fig. 1). The median follow-up time was 10 months (6–15 months). All patients included in the analysis were female, with 230 patients (54.8%) receiving trastuzumab and 190 patients (45.2%) receiving trastuzumab combined with pertuzumab. The median age was 52 years old (28–77 years old).The baseline clinical case characteristics of all patients were shown in Table 1. Of the 420 patients included in the analysis, 67 had a decrease of more than 10% in LVEF, and the incidence of cardiotoxicity was 15.9%. Most of the patients had an asymptomatic decrease, and all the patients had an LVEF of more than 50% before and after treatment. No patients were discontinued due to heart-related adverse reactions, and there were no congestive heart failure or drug-related deaths. The incidence of cardiotoxicity in trastuzumab group (H group) and trastuzumab combined with pertuzumab (HP group) for breast cancer was 14.3% and 17.9%, respectively (*P* > 0.05).

**Table 1:** Clinicopathological characteristics of 420 patients with early-stage breast cancer treated with anti-HER2-targeted therapy.

Characteristic	No. (%)	Characteristic	No. (%)
**Age, years**		**N stage**	
< 60	325 (77.4)	N0-1	165 (39.3)
≥ 60	95 (22.6)	N2-3	255 (60.7)
**BMI (kg/m**^**2**^**)**		**Tumor stage**	
< 24	197 (46.9)	I	50 (11.9)
≥ 24	223 (53.1)	II	291 (69.2)
**Menopausal status**		III	79 (18.8)
Premenopause	192 (45.7)	**HR status**	
Postmenopausal	228 (54.3)	Positive	341 (81.2)
**Hypertension**		Negative	79 (18.8)
Yes	98 (23.3)	**Ki67**	
No	322 (76.7)	< 20%	59 (14.0)
**Diabetes**		≥ 20%	361 (86.0)
Yes	52 (12.4)	**Neoadjuvant therapy**	
No	368 (87.6)	Yes	113 (26.9)
**CAD**		No	307 (73.1)
Yes	66 (15.7)	**Surgery type**	
No	354 (84.3)	Breast conserving	48 (11.4)
**Smoking**		Mastectomy	372 (88.6)
Yes	38 (9.0)	**Anthracyclines**	
No	382 (91.0)	Yes	294 (70.0)
**Invasive ductal carcinoma**		No	126 (30.0)
Yes	399 (95)	**Targeted therapy**	
No	21 (5)	H	230 (54.8)
**Histilogical grade**		HP	190 (45.2)
I	37 (8.8)	**Radiotherapy**	
II	302 (71.9)	Yes	360 (85.7)
III	81 (19.3)	No	60 (14.3)
**T stage**		**Radiotherapy position**	
T1–2	318 (75.7)	Left chest wall area	186 (51.7)
T3–4	102 (24.3)	Right chest wall area	174 (48.3)

*CAD* coronary artery disease, *H* trastuzumab, *HP* trastuzumab and pertuzumab.

Single factor analysis showed that age, hypertension, coronary heart disease, left chest wall radiotherapy and anthracene sequential therapy were the risk factors for the cardiotoxicity related to anti-HER2-targeted therapy in early breast cancer (*P* < 0.05). BMI (*P* = 0.147), menopausal status (*P* = 0.076), diabetes (*P* = 0.776), smoking (*P* = 0.977), histological grade (*P* = 0.998), T stage (*P* = 0.998) 0.184), N stage (*P* = 0.647), tumor stage (*P* = 0.760), HR status (*P* = 0.585), Ki-67 (*P* = 0.078), surgical method (*P* = 0.0.488), targeted therapy (*P* = 0.0.323), radiotherapy (*P* = 0.0.323) 0.0.550) had no significant correlation with cardiac toxicity, as shown in Table 2.

**Table 2:** Univariate analysis of cardiotoxicity associated with targeted therapy.

Characteristic	No cardiotoxicity n (%)	Cardiotoxicity n (%)	χ^2^	*P*
**Age, years**			9.834	0.002*
< 60	283 (87.1)	42 (12.9)		
≥ 60	70 (73.7)	25 (26.3)		
**BMI (kg/m**^**2**^**)**			2.100	0.147
< 24	171 (86.8)	26 (13.2)		
≥ 24	182 (81.6)	41 (18.4)		
**Menopausal status**			3.144	0.076
Premenopause	168 (87.5)	24 (12.5)		
Postmenopausal	185 (81.1)	43 (18.9)		
**Hypertension**			8.709	0.003*
Yes	73 (74.5)	25 (25.5)		
No	280 (87.0)	42 (13.0)		
**Diabetes**			0.081	0.776
Yes	43 (82.7)	9 (17.3)		
No	310 (84.2)	58 (15.8)		
**CAD**			14.702	< 0.001*
Yes	45 (68.2)	21 (31.8)		
No	308 (87.0)	46 (13.0)		
**Smoking**			0.001	0.977
Yes	32 (84.2)	6 (15.8)		
No	321 (84.0)	61 (16.0)		
**Invasive ductal carcinoma**			0.046	0.831
Yes	335 (84.0)	64 (16.0)		
No	18 (85.7)	3 (14.3)		
**Histilogical grade**			0.003	0.998
I	31 (83.8)	6 (16.2)		
II	254 (84.1)	48 (15.9)		
III	68 (84.0)	13 (16.0)		
**T stage**			1.762	0.184
T1–2	263 (82.7)	55 (17.3)		
T3–4	90 (88.2)	12 (11.8)		
**N stage**			0.210	0.647
N0–1	137 (83.0)	28 (17.0)		
N2–3	216 (84.7)	39 (15.3)		
**Tumor stage**			0.787	0.675
I	44 (88.0)	6 (12.0)		
II	242 (83.2)	49 (16.8)		
III	67 (84.8)	12 (15.2)		
**HR status**			0.299	0.585
Positive	285 (83.6)	56 (16.4)		
Negative	68 (86.1)	11 (13.9)		
**Ki67**			3.096	0.078
< 20%	45 (76.3)	14 (23.7)		
≥ 20%	308 (85.3)	53 (14.7)		
**Neoadjuvant therapy**			1.464	0.226
Yes	99 (87.6)	14 (12.4)		
No	254 (82.7)	53 (17.3)		
**Surgery type**			0.482	0.488
Breast conserving	42 (87.5)	6 (12.5)		
Mastectomy	311 (83.6)	61 (16.4)		
**Anthracyclines**			5.548	0.018*
Yes	239 (81.3)	55 (18.7)		
No	114 (90.5)	12 (9.5)		
**Targeted therapy**			0.976	0.323
H	197 (85.7)	33 (14.3)		
HP	156 (82.1)	34 (17.9)		
**Radiotherapy**			0.358	0.550
Yes	301 (83.6)	59 (16.4)		
No	52 (86.7)	8 (13.3)		
**Radiotherapy position**			8.978	0.003*
Left chest wall area	145 (78.0)	41 (22.0)		
Right chest wall area	156 (89.7)	18 (12.3)		

Significant values are in [bold].

*CAD* coronary artery disease, *H* trastuzumab, *HP* trastuzumab and pertuzumab.

*P < 0.05.

**Figure 2:**
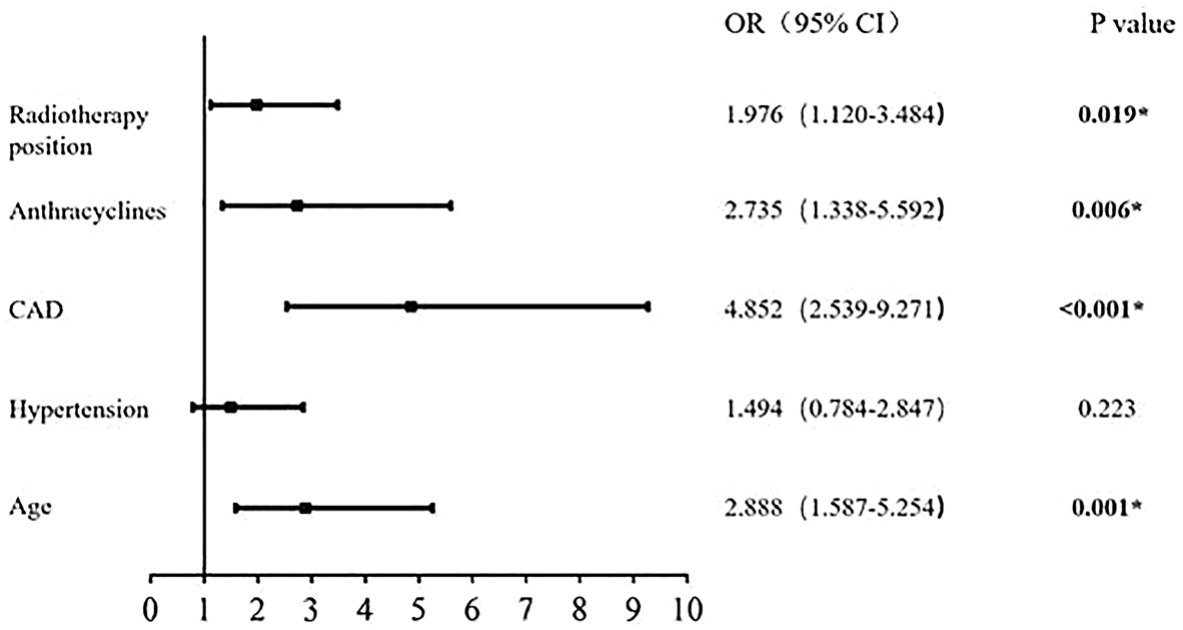
Multivariate analysis of cardiotoxicity associated with targeted therapy (*OR* odds ratio, *CAD* coronary artery disease; *P < 0.05).

Multivariate analysis of cardiotoxicity related to HER2-targeted therapy in early breast cancer showed that age, coronary heart disease, left chest wall radiotherapy, and anthracene sequential therapy were independent risk factors for cardiotoxicity (*P* < 0.05), (Fig. 2).

Comparison results of LVEF, hs-TnI and QTc between the non-cardiotoxic group and the cardiotoxic group at each time point are shown in Table 3.There was no significant difference in baseline LVEF and hs-TnI values between the two groups (*P* > 0.05), and there were significant differences in LVEF and hs-TnI values between the two groups 3, 6, 9 and 12 months (*P* < 0.05). There were significant differences in QTc between the two groups at baseline and 3, 6, 9 and 12 months (*P* < 0.05).

**Table 3:** Comparison of LVEF, hs-TnI and QTc at each time point between non-cardiotoxicity group and cardiotoxicity group.

Parameter	No cardiotoxicity n = 353	Cardiotoxicity n = 67	*P* value
**LVEF (%)**			
Baseline	65.07 ± 2.59	65.36 ± 1.94	0.404
3m	64.67 ± 2.99	63.45 ± 2.57	0.003*
6m	63.87 ± 3.99	61.93 ± 3.16	0.001*
9m	62.17 ± 4.11	59.65 ± 4.84	< 0.001*
12m	61.39 ± 4.01	56.44 ± 4.30	< 0.001*
**hsTnI (pg/ml)**			
Baseline	2.32 ± 3.03	2.61 ± 2.21	0.063
3m	2.33 ± 2.66	3.34 ± 2.69	0.012*
6m	2.41 ± 2.93	3.99 ± 3.16	0.001*
9m	2.78 ± 3.43	4.60 ± 3.40	< 0.001*
12m	2.85 ± 3.87	4.87 ± 3.61	< 0.001*
**QTc (ms)**			
Baseline	405.19 ± 22.27	415.86 ± 34.90	0.002*
3m	410.26 ± 28.94	430.63 ± 36.73	< 0.001*
6m	417.58 ± 37.43	444.42 ± 45.19	< 0.001*
9m	412.96 ± 29.89	440.26 ± 38.15	< 0.001*
12m	414.12 ± 26.97	438.85 ± 42.92	< 0.001*

Significant values are in [bold].

*m* month.

*P < 0.05.

The incidence of cardiotoxicity in H group and HP group was 14.3% and 17.9%, respectively (*P* > 0.05), and there was no significant difference in the incidence of cardiac toxicity between the two groups. Next, we conducted subgroup analysis for different risk factors, and compared whether there were significant differences in LVEF, hs-TnI and QTc at each time point between H group and HP group, so as to analyze whether HP dual target therapy increased the influence on the heart under different risk factors compared with H single target therapy.

### Subgroup analysis of LVEF in the two groups under different risk factors

In patients with CAD, HP group showed a more significant overall decline in LVEF compared with H group, and significant differences in LVEF between the two groups were observed at the 9th and 12th months (*P* < 0.05); in sequential treatment with anthracene, the decrease of LVEF was more significant between the two groups, significant differences were observed between the two groups at 6, 9, and 12 months (*P* < 0.05); however, there was no significant difference in LVEF between the two groups at each time point under the condition of age ≥ 60 and left chest wall radiotherapy (*P* > 0.05), as seen in Table 4.

**Table 4:** LVEF of patients in H group and HP group under various risk factors.

	H group	HP group	*P* value		H group	HP group	*P* value
	N = 55	N = 40			N = 49	N = 17	
**Age ≥ 60**				**CAD**			
Baseline	65.04 ± 2.03	64.68 ± 2.25	0.261	Baseline	64.88 ± 2.02	65.07 ± 1.54	0.725
3m	63.83 ± 2.13	63.68 ± 2.08	0.709	3m	63.21 ± 2.78	64.49 ± 2.31	0.252
6m	62.77 ± 3.42	62.74 ± 3.43	0.872	6m	61.44 ± 3.24	61.20 ± 3.62	0.683
9m	61.15 ± 3.58	61.77 ± 3.79	0.258	9m	60.73 ± 3.65	58.11 ± 3.97	0.037*
12m	58.89 ± 3.87	59.22 ± 3.46	0.237	12m	59.59 ± 3.89	56.82 ± 4.21	0.021*

	N = 91	N = 95			N = 184	N = 110	
**Left chest wall radiotherapy**				**Anthracyclines**			
Baseline	65.01 ± 2.58	65.13 ± 1.97	0.721	Baseline	64.73 ± 2.57	64.83 ± 2.97	0.619
3m	63.19 ± 3.17	63.28 ± 2.84	0.837	3m	63.25 ± 2.43	62.91 ± 2.85	0.207
6m	62.44 ± 3.95	62.52 ± 3.12	0.896	6m	62.83 ± 3.99	60.15 ± 3.62	< 0.001*
9m	61.29 ± 3.89	61.47 ± 3.71	0.623	9m	60.94 ± 3.63	59.12 ± 3.61	< 0.001*
12m	59.26 ± 4.45	59.08 ± 4.23	0.435	12m	59.36 ± 4.28	57.79 ± 4.35	< 0.001*

Significant values are in [bold].

*m* month.

*P < 0.05.

### Subgroup analysis of hs-TnI in the two groups under different risk factors

In patients with CAD, the overall trend of hs-TnI increase was more obvious in the HP group than in the H group, and there were significant differences in hs-TnI between the two groups at baseline and 3, 6, 9 and 12 months (*P* < 0.05); During sequential treatment of anthracene, the increase of hs-TnI was more obvious in the HP group than in the H group, and there were significant differences in hs-TnI between the two groups at baseline and 3, 6, 9 and 12 months (*P* < 0.05); however, there was no significant difference between the two groups in the age of 60 years and the left chest wall radiotherapy (*P* > 0.05); as seen in Table 5.

**Table 5:** hs-TnI of patients in H group and HP group under various risk factors.

	H group	HP group	*P* value		H group	HP group	*P* value
	N = 55	N = 40			N = 49	N = 17	
**Age ≥ 60**				**CAD**			
Baseline	2.24 ± 1.87	2.31 ± 2.63	0.248	Baseline	1.64 ± 1.37	3.18 ± 2.49	0.009*
3m	2.89 ± 2.11	2.87 ± 2.82	0.654	3m	1.90 ± 1.43	4.23 ± 2.86	< 0.001*
6m	3.24 ± 2.46	3.53 ± 3.49	0.473	6m	2.31 ± 1.28	4.96 ± 3.64	0.001*
9m	4.64 ± 3.23	3.59 ± 3.17	0.258	9m	2.71 ± 1.45	5.55 ± 3.66	< 0.001*
12m	4.43 ± 3.83	4.29 ± 3.65	0.755	12m	2.35 ± 1.27	6.64 ± 3.87	< 0.001*

	N = 91	N = 95			N = 184	N = 110	
**Left chest wall radiotherapy**				**Anthracyclines**			
Baseline	2.35 ± 1.59	2.22 ± 2.48	0.251	Baseline	2.52 ± 1.54	4.91 ± 3.83	0.043*
3m	2.97 ± 2.31	2.90 ± 2.95	0.629	3m	3.29 ± 2.37	5.56 ± 2.97	0.028*
6m	3.65 ± 2.75	3.43 ± 3.57	0.432	6m	3.96 ± 2.94	6.50 ± 3.74	0.006*
9m	4.53 ± 3.47	3.51 ± 3.26	0.205	9m	4.78 ± 3.55	7.34 ± 3.93	0.008*
12m	4.58 ± 3.77	4.38 ± 3.53	0.856	12m	4.93 ± 4.40	7.71 ± 3.70	0.001*

Significant values are in [bold].

*m* month.

*P < 0.05.

### Subgroup analysis of QTc in the two groups under different risk factors

In patients with CAD, the QTc of HP group showed a more obvious prolongation trend than that of H group, and there were significant differences in QTC between the two groups at 6, 9 and 12 months (*P* < 0.05); QTc prolongation in HP group was more obvious than that in H group at 3, 6, 9 and 12 months after sequential treatment (*P* < 0.05); There was no significant difference in QTc at each time point in patients aged ≥ 60 years and receiving left chest wall radiotherapy (*P* > 0.05); as seen in Table 6.

**Table 6:** QTc of patients in H group and HP group under various risk factors.

	H group	HP group	*P* value		H group	HP group	*P* value
	N = 55	N = 40			N = 49	N = 17	
**Age ≥ 60**				**CAD**			
Baseline	414.23 ± 27.68	420.88 ± 28.53	0.247	Baseline	412.78 ± 30.04	408.50 ± 30.35	0.086
3m	425.26 ± 30.92	436.52 ± 27.41	0.153	3m	434.38 ± 33.25	441.24 ± 31.92	0.059
6m	436.93 ± 33.21	442.21 ± 30.67	0.114	6m	427.78 ± 34.14	443.89 ± 38.37	0.001*
9m	434.37 ± 31.29	438.68 ± 26.54	0.274	9m	426.54 ± 31.38	448.26 ± 35.69	< 0.001*
12m	438.95 ± 28.47	443.68 ± 29.55	0.133	12m	430.12 ± 30.17	447.63 ± 34.42	< 0.001*

	N = 91	N = 95			N = 184	N = 110	
**Left chest wall radiotherapy**				**Anthracyclines**			
Baseline	409.11 ± 33.26	409.99 ± 31.47	0.844	Baseline	414.78 ± 33.89	416.79 ± 30.73	0.417
3m	424.79 ± 36.85	426.52 ± 32.08	0.326	3m	426.32 ± 35.94	437.81 ± 32.68	< 0.001*
6m	437.45 ± 35.93	438.57 ± 38.81	0.613	6m	431.67 ± 31.77	451.85 ± 29.61	< 0.001*
9m	428.12 ± 38.26	431.91 ± 41.22	0.109	9m	419.17 ± 37.26	443.47 ± 31.87	< 0.001*
12m	431.88 ± 40.32	436.44 ± 39.14	0.095	12m	420.84 ± 38.28	448.24 ± 34.92	< 0.001*

Significant values are in [bold].

*m* month.

*P < 0.05.

### Sensitivity and specificity of hs-TnI and QTc

**Figure 3:**
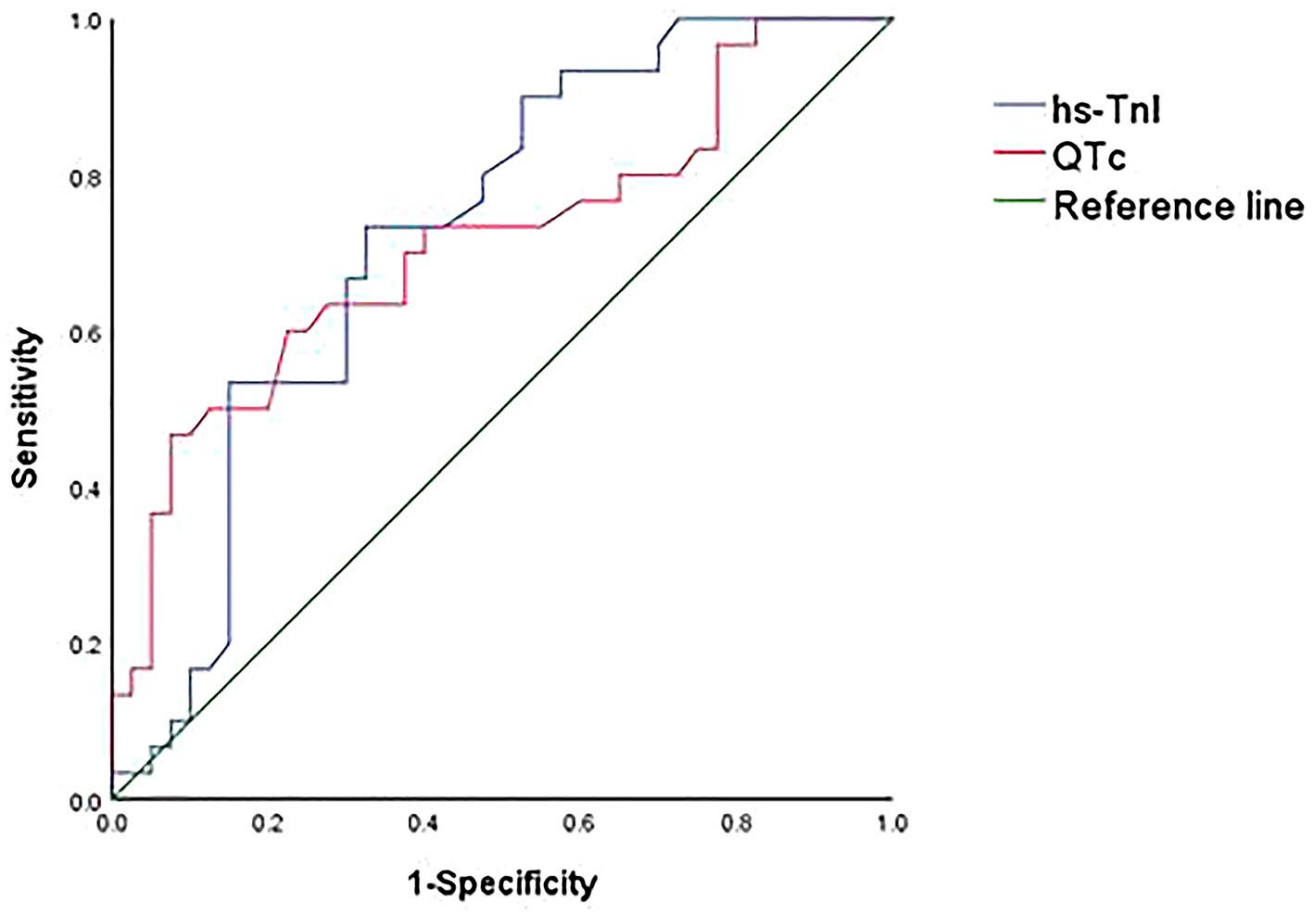
ROC curves of hs-TnI and QTc for the diagnosis of cardiotoxicity.

According to the above research results, we found that the changes of hs-TNI and QTc were closely related to the changes of LVEF, and even showed significant changes prior to LVEF in some conditions. In order to further verify the sensitivity and specificity of hs-TnI and QTc on the cardiotoxicity related to anti-HER2 targeted therapy in breast cancer, we selected the parameters at the end of treatment, the 12th month, for ROC curve prediction analysis. The results showed that the areas under the ROC curve of hs-TnI and QTc were 0.724 and 0.713, respectively, which had a statistical difference compared with 0.5 (*P* < 0.05), indicating that hs-TnI and QTc have a certain predictive effect on the cardiotoxicity related to anti-HER2 targeted therapy in breast cancer (Table 7, Fig. 3).

**Table 7:** ROC curve analysis of hs-TnI and QTc for the diagnosis of cardiotoxicity.

Variables	Cutoff value	Sensitivity	1-Specificity	AUC	95% CI	*P* value
Hs-TnI (pg/ml)	3.35	0.733	0.325	0.724	0.605–0.843	0.001
QTc (ms)	434.5	0.467	0.075	0.713	0.588–0.839	0.002

The cutoff values are the parameter value corresponding to the maximum value obtained by subtracting sensitivity − (1-specificity), at this threshold, the value of the Yoden index is the highest, that means, the highest accuracy of disease diagnosis.

*CI* confidence interval, *ROC* receiver–operator characteristic.

## Discussion

Trastuzumab and pertuzumab are monoclonal antibodies for anti-HER2 therapy. They have complementary effects in mechanism, and combined administration can achieve a more complete blocking effect on the HER2 pathway^4^. Some studies have shown that monoclonal antibodies are associated with an increased risk of cardiotoxicity, especially when combined with anthracyclines^17–21^. However, autophagy disorders in cardiomyocytes have been identified as another potential mechanism for cardiotoxicity caused by trastuzumab rather than pertuzumab recently^28^. This may provide further understanding and support for the mechanism by which the addition of pertuzumab in the course of anti-HER2-targeted therapy does not increase cardiac toxicity.

In our study, a total of 420 patients with HER2-positive early-stage breast cancer received targeted therapy for more than half a year were eventually included to evaluate the cardiac safety of anti-HER2-targeted therapy. Studies have reported that trastuzumab related cardiotoxicity usually occurs in the median of 5–6 months^21,29,30^, therefore, we selected patients who received targeted therapy for more than 6 months for the study. The incidence of cardiotoxicity in this study was 15.9%, consistent with clinical reports of cardiotoxicity associated with anti-HER2 targeting (5–19%)^13–16^. All the patients had LVEF above 50% before and after treatment. During the follow-up, no patients were discontinued due to heart-related adverse reactions, and no patients had congestive heart failure or drug-related death. From the perspective of cardiac safety, anti-HER2-targeted therapy for breast cancer was well tolerated. Similar results were found in BERENICE, KRISTINE, TRAIN-2 studies, which all supported that trastuzumab combined with pertuzumab did not significantly increase cardiotoxicity^31–33^. In the NeoSphere and CLEOPATRA study, more than 90% of patients treated with trastuzumab and pertuzumab did not develop any grade of left ventricular dysfunction^34,35^. In addition, some studies have shown that trastuzumab targeted therapy is a safe treatment for most patients from a cardiac perspective with long-term follow-up^36–38^. In this study, the incidence of cardiotoxicity in H group and HP group was 14.3% and 17.9%, respectively (*P* > 0.05), suggesting the HP treatment has not increased in patients with cardiac toxicity observably, but in the anthracene sequential treatment group, the effects of trastuzumab and pertuzumab on cardiac function related indexes LVEF, hs-TnI and QTc were more obvious, suggesting that anthracene sequential therapy increased the effects of trastuzumab and pertuzumab on the heart.

Conclusions on the risk factors of cardiotoxicity associated with targeted therapy are mixed. In a German prospective study of 3940 patients and NSABP B-31, N9831 combined analysis, older age (> 50 years old) was a risk factor for trastuzumab-related cardiotoxicity, and another retrospective study showed that age of > 65 years old was a risk factor^39,40^. A multicenter study found that hypertension is a potential risk factor for trastuzumab-related cardiotoxicity^41^. Two other retrospective studies concluded that radiotherapy and obesity were high risk factors for trastuzumab-related cardiotoxicity^42,43^. Other studies have found that CAD, diabetes, dyslipidemia, smoking, anthracycline sequential therapy are anti-HER2 targeted therapy-related risk factors for cardiac toxicity^44–47^. In addition to age, CAD, left chest wall radiotherapy, and anthracene sequential therapy were found to be independent risk factors for cardiotoxicity associated with targeted therapy for breast cancer in this study. Therefore, when anti-HER2-targeted therapy is applied in clinical practice, the changes of LVEF, hs-TnI and QTc in patients with age ≥ 60 years old, CAD, left chest wall radiotherapy and sequential anthracene therapy should be closely watched.

LVEF is often used to diagnose cardiotoxicity in patients who may have cardiotoxicity in the course of antitumor therapy, but it is not sensitive to reflect the early heart injury. Echography is a more common method for LVEF assessment, which is more economical and convenient. MUGA also can be used for assessing LVEF but not every patient used this method. Cardiac biomarkers, as a new detection method, have been widely studied for early detection, evaluation and monitoring of cardiotoxicity. Results of a small multicenter cohort of 78 patients showed that elevated hs-TnI was associated with cardiotoxicity induced by anthracycline and trastuzumab, and suggested a close relationship between elevated hs-TnI and subsequent cardiac dysfunction^48^. In addition, three studies have suggested that hs-TnI can predict the cardiotoxicity associated with anthracycline and trastuzumab, but more evidence is needed to support the importance of evaluating drug-induced cardiac injury over time^49–51^. In contrast, a prospective study showed that hs-TnI levels were detectable in patients with early-stage breast cancer treated with anthracycline and trastuzumab during treatment, however, they were not associated with asymptomatic decreased LVEF and did not predict cardiotoxicity^52^. Based on the inconsistent conclusions reported in the above literatures, we collected the hs-TnI of the enrolled patients at each time point and conducted statistical analysis. The results showed that there was a significant correlation between hs-TnI and the cardiotoxicity related to anti-HER2 targeted therapy. There were significant differences in hs-TnI values between the cardiotoxic group and non-cardiotoxic group at 3, 6, 9 and 12 months (*P* < 0.05), suggesting that hs-TnI is closely related to the cardiotoxicity associated with anti-HER2-targeted therapy. Moreover, in the subgroup analysis, in the case of CAD, significant changes in LVEF were observed at 9 and 12 months after treatment in the HP group compared to the H group, while significant differences in hs-TnI were observed in the two groups from baseline. In the case of sequential anthracene treatment, the same is true. Both groups showed significant changes in hs-TnI before LVEF, suggesting that in the course of anti-HER2-targeted therapy, hs-TnI may be earlier than the changes in LVEF in some cases. Furthermore, the ROC curve prediction analysis also further supported the predictive value of hs-TnI in predicting the cardiotoxicity related to anti-HER2-targeted therapy in breast cancer. It is also suggested that early attention should be paid to the changes of hs-TnI in patients with CAD and sequential treatment of anthracene in the application of trastuzumab and pertuzumab in clinical practice.

Prolongation of QTc means delayed repolarization of the heart, which creates an electrophysiological environment conducive to the development of ventricular arrhythmias, most notably Torsade de Pointes (TDP), in severe cases, ventricular arrhythmias can be induced and even lead to sudden death^53^. Data have shown that tyrosinase inhibitors such as lapatinib cause QTC prolongation^54^. Monoclonal antibodies are considered to have less impact on QTc due to their larger molecules, which cannot directly enter the binding site of the drug channel, and their higher targeting specificity compared with small-molecule drugs^55^. Notably, anthracyclines that are often used in combination with monoclonal antibodies have been shown to be associated with prolonged QTc or other arrhythmias^56,57^. In this study, there were significant differences in QTc at each time point between the cardiotoxic group and the non-cardiotoxic group (*P* < 0.05), suggesting a significant correlation between QTc prolongation and cardiotoxicity associated with anti-HER2-targeted therapy. In addition, in the subgroup analysis, QTc of the two groups also showed significant changes earlier than LVEF in the case of coronary heart disease and sequential treatment of anthracene, suggesting that the change of QTc may be earlier than LVEF in some cases during anti-HER2-targeted therapy. Moreover, ROC curve prediction analysis also further verified the predictive value of QTc on the cardiotoxicity related to anti-HER2 targeted therapy in breast cancer.

This study holdings a few limitations. This is a retrospective study, possibly leading to selection bias. When using echocardiography to measure parameters, subjective differences among physicians are inevitable. Therefore, prospective studies should be conducted in the future to continue to expand the sample size and reduce the subjective differences among physicians.

## Conclusion

In summary, the cardiac safety of anti-HER2-targeted therapy for early breast cancer was encouraging. Trastuzumab and pertuzumab did not significantly increase cardiotoxicity compared with trastuzumab. Age, CAD, left chest wall radiotherapy, and anthracene sequential therapy were independent risk factors for the cardiotoxicity associated with anti-HER2-targeted therapy in early breast cancer. Subgroup analysis suggests that in the presence of CAD and sequential treatment with anthracene, trastuzumab and pertuzumab may increase the cardiac burden compared with trastuzumab. Hs-TnI and QTc have certain predictive value for the cardiotoxicity related to anti-HER2 targeted therapy in breast cancer.

## Data Availability

The datasets used during the present study are available from the corresponding author upon reasonable request.
